# Supplementary material for: Prospective observational study of oxidative stress in the pathology of benign prostatic hyperplasia with bladder diverticulum
Source: PLoS One. 2025 May 15;20(5):e0323677. doi: 10.1371/journal.pone.0323677 (PMC12080795; doi:10.1371/journal.pone.0323677)
Supplement: S1 Table — (DOCX) [file pone.0323677.s001.docx]

Table S1. Oxidative stress in BPH.

| Author et al., Year, | Oxidative stress biomarkers and their actual values |
| --- | --- |
| Jin et al., 2024^1^ | AR and NOX4-mediated oxidative stress upregulated in BPH; Apocynin treatment reduced oxidative stress. |
| Ohtake et al., 2018^2^ | 8-OHdG levels in BPH: 71.4% moderate expression, 28.6% strong expression. |
| Vital et al., 2016^3^ | 8-OHdG levels significantly higher in BPH tissues; levels correlated with prostate weight. |
| Pace et al., 2010^4^ | High plasma peroxides and decreased TEAC levels in BPH patients; no significant changes in SOD activity. |
| Ahmad et al., 2012^5^ | Antioxidant enzyme activities and GSH levels decreased; lymphocytic DNA damage increased in BPH patients. |
| Akanni et al., 2020^6^ | In BPH rats, MDA increased by 97%, SOD increased by 25%. |
| Al-Barzinj, 2020^7^ | In BPH patients who smoke, MDA levels increased compared to non-smokers. |
| Atawia et al., 2013^8^ | Silymarin restored oxidative status to normal in prostate tissues in BPH rats. |
| Aydin et al., 2006^9^ | In BPH patients, TBARS levels increased, SOD activity decreased, Zn levels decreased compared to controls. |
| Calo et al., 2006^10^ | Doxazosin reduced oxidative stress-related proteins p22(phox) and HO-1 in BPH patients. |
| Chang et al., 2018^11^ | MDA levels significantly higher in BPH patients; 8-OHdG and DNA strand breakage highest in BPH patients compared to controls. |
| Chen et al., 2010^12^ | In BPH patients undergoing TURP, MDA increased and TOA decreased; higher MDA and lower TOA with distilled water irrigant. |
| Choi et al., 2024^13^ | HLT-101 decreased ROS levels and oxidative stress via Nrf-2/HO-1 activation in BPH models. |
| Cimino et al., 2014^14^ | TTG levels in BPH patients: 34 nmol RSH/mg protein; higher than in PCa patients (1.1 nmol RSH/mg protein). |
| Colado-Velazquez et al., 2015^15^ | Serenoa repens decreased MDA levels; increased GSH, SOD, and catalase activity in obese rats with BPH. |
| Colado-Velazquez et al., 2023^16^ | In BPH rats, obesity increased MDA, decreased SOD and catalase; STE and SR treatments reversed these effects. |
| Dearakhshandeh et al., 2019^17^ | In BPH-induced dogs, GPX and SOD levels decreased; MDA increased slightly. |
| Domoslawska et al., 2022^18^ | In BPH dogs, serum TAC significantly lower (3.10 ± 0.56 vs. 4.20 ± 1.60 μmol/g protein); no significant differences in protein and lipid oxidation biomarkers. |
| El-Sherbiny et al., 2021^19^ | Diacerein treatment increased prostatic GSH, SOD, and CAT activities; reduced lipid peroxidation levels in BPH rats. |
| Elsherbini et al., 2022^20^ | In BPH rats, MDA levels increased; SOD, CAT, and TAC activities decreased; O. majorana treatment restored antioxidant activities. |
| Ercan et al., 2019^21^ | In BPH patients, 8-OHdG levels increased compared to controls; after surgery, 8-OHdG and SOD activity decreased, CoQ10 increased. |
| Hsu et al., 2021^22^ | Phloretin treatment reduced MDA levels; increased SOD and GPx activities in BPH rats. |
| Kaya et al., 2017^23^ | In BPH patients, MDA levels increased; CAT activity increased; trace element levels decreased compared to controls. |
| Li et al., 2019^24^ | In high-fat diet-induced BPH rats, MDA increased; GSH-Px, GR, GSH, and SOD decreased. |
| Matsumoto et al., 2010^25^ | Eviprostat treatment decreased urinary 8-OHdG levels by 2.5-fold in BPH patients. |
| Savas et al., 2009^26^ | No significant difference in TAC, TOS, OSI between BPH patients and controls. |
| Semenov et al., 2021^27^ | Astaxanthin treatment normalized SOD activity in BPH rats. |
| Yang et al., 2014^28^ | AHT and TFA treatment increased SOD, GPx, CAT activities; decreased MDA levels in BPH rats. |

**References**

1. Jin B-R, Kim H-J, Na J-H, Lee W-K, An H-J. Targeting benign prostate hyperplasia treatments: AR/TGF-β/NOX4 inhibition by apocynin suppresses inflammation and proliferation. *Journal of Advanced Research*. 2024;57:135-147.

2. Ohtake S, Kawahara T, Ishiguro Y, et al. Oxidative stress marker 8-hydroxyguanosine is more highly expressed in prostate cancer than in benign prostatic hyperplasia. *Mol Clin Oncol*. Sep 2018;9(3):302-304. doi:10.3892/mco.2018.1665

3. Vital P, Castro P, Ittmann M. Oxidative stress promotes benign prostatic hyperplasia. *Prostate*. Jan 2016;76(1):58-67. doi:10.1002/pros.23100

4. Pace G, Di Massimo C, De Amicis D, et al. Oxidative stress in benign prostatic hyperplasia and prostate cancer. *Urol Int*. 2010;85(3):328-33. doi:10.1159/000315064

5. Ahmad M, Suhail N, Mansoor T, Banu N, Ahmad S. Evaluation of oxidative stress and DNA damage in benign prostatic hyperplasia patients and comparison with controls. *Indian J Clin Biochem*. Oct 2012;27(4):385-8. doi:10.1007/s12291-012-0229-4

6. Akanni OO, Owumi SE, Olowofela OG, Adeyanju AA, Abiola OJ, Adaramoye OA. Protocatechuic acid ameliorates testosterone-induced benign prostatic hyperplasia through the regulation of inflammation and oxidative stress in castrated rats. *J Biochem Mol Toxicol*. Aug 2020;34(8):e22502. doi:10.1002/jbt.22502

7. Al-Barzinj R. Estimation levels of prostate-specific antigen, interleukin-8, oxidative stress and some inflammatory markers in sera of benign prostatic hyperplasia patients who have smoking habits as a risk factor. *Cell Mol Biol (Noisy-le-grand)*. Oct 31 2020;66(7):124-130.

8. Atawia RT, Tadros MG, Khalifa AE, Mosli HA, Abdel-Naim AB. Role of the phytoestrogenic, pro-apoptotic and anti-oxidative properties of silymarin in inhibiting experimental benign prostatic hyperplasia in rats. *Toxicol Lett*. May 23 2013;219(2):160-9. doi:10.1016/j.toxlet.2013.03.002

9. Aydin A, Arsova-Sarafinovska Z, Sayal A, et al. Oxidative stress and antioxidant status in non-metastatic prostate cancer and benign prostatic hyperplasia. *Clin Biochem*. Feb 2006;39(2):176-9. doi:10.1016/j.clinbiochem.2005.11.018

10. Calo LA, Pagnin E, Davis PA, et al. Effect of doxazosin on oxidative stress-related proteins in benign prostatic hyperplasia. *Urol Int*. 2006;76(1):36-41. doi:10.1159/000089733

11. Chang WH, Lee CC, Yen YH, Chen HL. Oxidative damage in patients with benign prostatic hyperplasia and prostate cancer co-exposed to phthalates and to trace elements. *Environ Int*. Dec 2018;121(Pt 2):1179-1184. doi:10.1016/j.envint.2018.10.034

12. Chen SS, Wu SB, Wei YH. The difference in oxidative stress of the blood between using 5% glucose water and distilled water as the irrigant for BPH patients undergoing transurethral resection of the prostate. *World J Urol*. Feb 2010;28(1):33-7. doi:10.1007/s00345-009-0438-5

13. Choi YJ, Wedamulla NE, Kim SH, et al. Salvia miltiorrhiza Bunge Ameliorates Benign Prostatic Hyperplasia through Regulation of Oxidative Stress via Nrf-2/HO-1 Activation. *J Microbiol Biotechnol*. May 28 2024;34(5):1059-1072. doi:10.4014/jmb.2308.08053

14. Cimino S, Favilla V, Russo GI, et al. Oxidative stress and body composition in prostate cancer and benign prostatic hyperplasia patients. *Anticancer Res*. Sep 2014;34(9):5051-6.

15. Colado-Velazquez J, III, Mailloux-Salinas P, Medina-Contreras J, Cruz-Robles D, Bravo G. Effect of Serenoa Repens on Oxidative Stress, Inflammatory and Growth Factors in Obese Wistar Rats with Benign Prostatic Hyperplasia. *Phytother Res*. Oct 2015;29(10):1525-31. doi:10.1002/ptr.5406

16. Colado-Velazquez JI, Mailloux-Salinas P, Arias-Chavez DJ, et al. Lipidic extract of whole tomato reduces hyperplasia, oxidative stress and inflammation on testosterone-induced BPH in obese rats. *Int Urol Nephrol*. Mar 2023;55(3):529-539. doi:10.1007/s11255-022-03383-2

17. Dearakhshandeh N, Mogheiseh A, Nazifi S, Ahrari Khafi MS, Abbaszadeh Hasiri M, Golchin-Rad K. Changes in the oxidative stress factors and inflammatory proteins following the treatment of BPH-induced dogs with an anti-proliferative agent called tadalafil. *J Vet Pharmacol Ther*. Nov 2019;42(6):665-672. doi:10.1111/jvp.12805

18. Domoslawska A, Zdunczyk S, Kankofer M, Bielecka A. Oxidative stress biomarkers in dogs with benign prostatic hyperplasia. *Ir Vet J*. Dec 15 2022;75(1):21. doi:10.1186/s13620-022-00228-3

19. El-Sherbiny M, El-Shafey M, El-Din El-Agawy MS, Mohamed AS, Eisa NH, Elsherbiny NM. Diacerein ameliorates testosterone-induced benign prostatic hyperplasia in rats: Effect on oxidative stress, inflammation and apoptosis. *Int Immunopharmacol*. Nov 2021;100:108082. doi:10.1016/j.intimp.2021.108082

20. Elsherbini DMA, Almohaimeed HM, El-Sherbiny M, et al. Origanum majorana L. Extract Attenuated Benign Prostatic Hyperplasia in Rat Model: Effect on Oxidative Stress, Apoptosis, and Proliferation. *Antioxidants (Basel)*. Jun 11 2022;11(6)doi:10.3390/antiox11061149

21. Ercan M, Alp HH, Kocaturk H, Bakan N, Gul M. Oxidative stress before and after surgery in benign prostatic hyperplasia patients. *Andrologia*. Sep 2019;51(8):e13326. doi:10.1111/and.13326

22. Hsu CY, Lin YS, Weng WC, et al. Phloretin Ameliorates Testosterone-Induced Benign Prostatic Hyperplasia in Rats by Regulating the Inflammatory Response, Oxidative Stress and Apoptosis. *Life (Basel)*. Jul 26 2021;11(8)doi:10.3390/life11080743

23. Kaya E, Ozgok Y, Zor M, et al. Oxidative stress parameters in patients with prostate cancer, benign prostatic hyperplasia and asymptomatic inflammatory prostatitis: A prospective controlled study. *Adv Clin Exp Med*. Oct 2017;26(7):1095-1099. doi:10.17219/acem/66837

24. Li Y, Shi B, Dong F, Zhu X, Liu B, Liu Y. Effects of inflammatory responses, apoptosis, and STAT3/NF-kappaB- and Nrf2-mediated oxidative stress on benign prostatic hyperplasia induced by a high-fat diet. *Aging (Albany NY)*. Aug 14 2019;11(15):5570-5578. doi:10.18632/aging.102138

25. Matsumoto S, Hanai T, Matsui T, Oka M, Tanaka M, Uemura H. Eviprostat suppresses urinary oxidative stress in a rabbit model of partial bladder outlet obstruction and in patients with benign prostatic hyperplasia. *Phytother Res*. Feb 2010;24(2):301-3. doi:10.1002/ptr.2909

26. Savas M, Verit A, Ciftci H, et al. Oxidative Stress in BPH. *JNMA J Nepal Med Assoc*. Jan-Mar 2009;48(173):41-5.

27. Semenov AL, Gubareva EA, Ermakova ED, et al. Astaxantin and Isoflavones Inhibit Benign Prostatic Hyperplasia in Rats by Reducing Oxidative Stress and Normalizing Ca/Mg Balance. *Plants (Basel)*. Dec 12 2021;10(12)doi:10.3390/plants10122735

28. Yang X, Yuan L, Xiong C, Yin C, Ruan J. Abacopteris penangiana exerts testosterone-induced benign prostatic hyperplasia protective effect through regulating inflammatory responses, reducing oxidative stress and anti-proliferative. *J Ethnopharmacol*. Nov 18 2014;157:105-13. doi:10.1016/j.jep.2014.09.025
