# Supplementary material for: Prospective observational study of oxidative stress in the pathology of benign prostatic hyperplasia with bladder diverticulum
Source: PLoS One. 2025 May 15;20(5):e0323677. doi: 10.1371/journal.pone.0323677 (PMC12080795; doi:10.1371/journal.pone.0323677)
Supplement: S2 Table — (DOCX) [file pone.0323677.s002.docx]

Table S2. Oxidative stress in bladder diverticulum and cancer.

| Author et al., Year | Oxidative stress biomarkers |
| --- | --- |
| Abdelrahman et al., 2023^1^ | Vinpocetine (30 mg/kg) reduced iNOS, TNF-alpha, and Bax expression; increased total antioxidant capacity (TAC) in bladder tissue of mice (actual values not provided). |
| Ali-El-Dein et al., 2024^2^ | In bladder cancer tissues, increased levels of MDA, 8-OHdG, 3-NT, AGEs, AOPP, ROS; decreased SOD2 levels associated with heavy metals (actual values not provided). |
| Darzi et al., 2024^3^ | After BCG therapy, MDA levels decreased, TAC levels increased in bladder cancer patients (actual values not provided). |
| Dong et al., 2024^4^ | Developed a prognostic model based on 4 oxidative stress-related genes (RBPMS, CRYAB, P4HB, PDGFRA); no actual biomarker values provided. |
| El-Agrody et al., 2024^5^ | In bladder cancer tissues, MDA levels significantly higher, SOD activity lower compared to non-cancerous tissues (actual values not provided). |
| Fan et al., 2024^6^ | Ivermectin induced ROS production and DNA damage in bladder cancer cells (actual values not provided). |
| Fang et al., 2024^7^ | In IC/BPS patients, NRF2 expression increased, GPX4 decreased; Wnt/beta-catenin signaling reduced ROS and inhibited ferroptosis (actual values not provided). |
| Galiniak et al., 2023^8^ | Serum and urine levels of AOPP, Amadori products, TAC, TOS, OSI, and MDA elevated in bladder cancer patients (actual values not provided). |
| Geng et al., 2024^9^ | H2O2-induced oxidative stress increased TGF-beta2 expression and EMT markers in bladder smooth muscle cells (actual values not provided). |
| Golmohammadi et al., 2023^10^ | Quercetin impacts bladder cancer by affecting oxidative stress pathways; no actual biomarker values provided. |
| Huang et al., 2024^11^ | Developed risk-stratification model based on oxidative stress-related genes in bladder cancer; no actual biomarker values provided. |
| Jiang et al., 2023^12^ | Elevated urinary levels of 8-isoprostane, 8-OHdG, and TAC in DO, IC/BPS, and DV cases; HSB cases with low TAC (actual values not provided). |
| Nabieh et al., 2023^13^ | In boys with neuropathic bladder and posterior urethral valve: increased blood levels of Cu, Pb, CAT, MDA, GSH; decreased Zn compared to controls (actual values not provided). |
| Saima et al., 2023^14^ | Caftaric acid treatment increased SOD by 93%, CAT by 92%, GSH by 90%; decreased iNOS by 97%, IL-6 by 90%, TGF-beta1 by 83%, TNF-alpha by 96% compared to diseased group. |
| Singh et al., 2023^15^ | In NMIBC patients, MDA, NO, and SOD levels were significantly higher in BCG-nonresponsive group than in BCG-responsive group (actual values not provided). |
| Wang L. et al., 2024^16^ | In Nrf2 knockout diabetic mice, bladder AGEs, ROS, and MDA levels increased; SOD and GSH levels decreased (actual values not provided). |
| Wu et al., 2024^17^ | High-glucose induced upregulation of AIF-1 increased IL-6, TNF-alpha, and ROS levels in bladder urothelium (actual values not provided). |
| Ye et al., 2023^18^ | HMGB1 increased urothelial oxidative stress markers 4HNE and phospho-ERK1/2 staining (actual values not provided). |
| Zhang et al., 2024^19^ | Inhibition of KDM4A led to decreased SQLE transcription, squalene accumulation, induced ROS clearance, suppressed JNK/c-Jun phosphorylation (actual values not provided). |

**References**

1. Abdelrahman RS, Nashar EME, Alghamdi MA, Al-Khater KM, Taha RI. Phosphodiesterase1 inhibitor "Vinpocetine" ameliorates the inflammation, apoptosis and oxidative stress induced by cyclophosphamide in urinary bladder: an experimental study. *Int Urol Nephrol*. Jan 2023;55(1):129-139. doi:10.1007/s11255-022-03246-w

2. Ali-El-Dein B, Abdelgawad M, Tarek M, et al. Bladder cancer associated with elevated heavy metals: Investigation of probable carcinogenic pathways through mitochondrial dysfunction, oxidative stress and mitogen-activated protein kinase. *Urol Oncol*. Oct 7 2024;doi:10.1016/j.urolonc.2024.09.009

3. Darzi MM, Neamati N, Sadeghi F, Bijani A, Moudi E. An Intricate Relationship Between miR-155-5p Expression and Oxidative Stress in Bladder Cancer Patients Treated with Calmette-Guerin Immunotherapy. *Int J Mol Cell Med*. 2024;13(2):186-197. doi:10.22088/IJMCM.BUMS.13.2.186

4. Dong Y, Wu X, Xu C, et al. Prognostic model development and molecular subtypes identification in bladder urothelial cancer by oxidative stress signatures. *Aging (Albany NY)*. Feb 1 2024;16(3):2591-2616. doi:10.18632/aging.205499

5. El-Agrody E, Abol-Enein H, Mortada WI, Awadalla A, Tarabay HH, Elkhawaga OA. Does the Presence of Heavy Metals Influence the Gene Expression and Oxidative Stress in Bladder Cancer? *Biol Trace Elem Res*. Aug 2024;202(8):3475-3482. doi:10.1007/s12011-023-03950-3

6. Fan N, Zhang L, Wang Z, Ding H, Yue Z. Ivermectin Inhibits Bladder Cancer Cell Growth and Induces Oxidative Stress and DNA Damage. *Anticancer Agents Med Chem*. 2024;24(5):348-357. doi:10.2174/0118715206274095231106042833

7. Fang W, Song X, Li H, et al. Wnt/beta-catenin signaling inhibits oxidative stress-induced ferroptosis to improve interstitial cystitis/bladder pain syndrome by reducing NF-kappaB. *Biochim Biophys Acta Mol Cell Res*. Oct 2024;1871(7):119766. doi:10.1016/j.bbamcr.2024.119766

8. Galiniak S, Molon M, Biesiadecki M, Mokrzynska A, Balawender K. Oxidative Stress Markers in Urine and Serum of Patients with Bladder Cancer. *Antioxidants (Basel)*. Jan 26 2023;12(2)doi:10.3390/antiox12020277

9. Geng J, Zhang X, Zhang Y, et al. TGFbeta2 mediates oxidative stress-induced epithelial-to-mesenchymal transition of bladder smooth muscle. *In Vitro Cell Dev Biol Anim*. Aug 2024;60(7):793-804. doi:10.1007/s11626-024-00864-9

10. Golmohammadi M, Elmaghraby DA, Ramirez-Coronel AA, et al. A comprehensive view on the quercetin impact on bladder cancer: Focusing on oxidative stress, cellular, and molecular mechanisms. *Fundam Clin Pharmacol*. Oct 2023;37(5):900-909. doi:10.1111/fcp.12896

11. Huang J, Zhou D, Luo W, Liu Y, Zheng H, Wang Y. Integrating oxidative-stress biomarkers into a precision oncology risk-stratification model for bladder cancer prognosis and therapy. *Front Cell Dev Biol*. 2024;12:1453448. doi:10.3389/fcell.2024.1453448

12. Jiang YH, Jhang JF, Kuo HC. Urinary Oxidative Stress Biomarker Levels Might Be Useful in Identifying Functional Bladder Disorders in Women with Frequency and Urgency Syndrome. *J Clin Med*. Mar 17 2023;12(6)doi:10.3390/jcm12062336

13. Nabieh KA, Helmy TE, Abou El-Reash YG, Mortada WI. Relation between blood levels of heavy metals and some markers of oxidative stress among boys with neuropathic bladder and posterior urethral valve. *J Trace Elem Med Biol*. Mar 2023;76:127123. doi:10.1016/j.jtemb.2022.127123

14. Saima, Anjum I, Najm S, et al. Caftaric Acid Ameliorates Oxidative Stress, Inflammation, and Bladder Overactivity in Rats Having Interstitial Cystitis: An In Silico Study. *ACS Omega*. Aug 8 2023;8(31):28196-28206. doi:10.1021/acsomega.3c01450

15. Singh V, Singh MK, Jain M, Pandey AK, Kumar A, Sahu DK. The relationship between BCG immunotherapy and oxidative stress parameters in patients with nonmuscle invasive bladder cancer. *Urol Oncol*. Dec 2023;41(12):486 e25-486 e32. doi:10.1016/j.urolonc.2023.09.008

16. Wang L, Sun W, Ren G, et al. Deletion of Nrf2 induced severe oxidative stress and apoptosis in mice model of diabetic bladder dysfunction. *Int Urol Nephrol*. Oct 2024;56(10):3231-3240. doi:10.1007/s11255-024-04064-y

17. Wu Q, Qin B, Wu X, et al. Allograft inflammatory factor-1 enhances inflammation and oxidative stress via the NF-kappaB pathway of bladder urothelium in diabetic rat model. *Cytokine*. Jan 2024;173:156438. doi:10.1016/j.cyto.2023.156438

18. Ye S, Mahmood DFD, Ma F, Leng L, Bucala R, Vera PL. Urothelial Oxidative Stress and ERK Activation Mediate HMGB1-Induced Bladder Pain. *Cells*. May 22 2023;12(10)doi:10.3390/cells12101440

19. Zhang J, Xu H, He Y, et al. Inhibition of KDM4A restricts SQLE transcription and induces oxidative stress imbalance to suppress bladder cancer. *Redox Biol*. Oct 22 2024;77:103407. doi:10.1016/j.redox.2024.103407
