# Supplementary material for: Prospective observational study of oxidative stress in the pathology of benign prostatic hyperplasia with bladder diverticulum
Source: PLoS One. 2025 May 15;20(5):e0323677. doi: 10.1371/journal.pone.0323677 (PMC12080795; doi:10.1371/journal.pone.0323677)
Supplement: Attachment — Submitted filename: Response to Reviewers.docx [file pone.0323677.s004.docx]

Manuscript No. PONE-D-24-50162

Title: Prospective observational study of oxidative stress in the pathology of benign prostatic hyperplasia with bladder diverticulum

Journal PLOS ONE

**Dear Professor Wroński and Reviewer,**

Many thanks for your critical strategic comments, which have significantly improved the present manuscript quality. We carefully revise our paper according to these important questions, and answered then in a point-to-point way as follows:

**Comments from Academic Editor Professor Stanisław Wroński**

Q: After careful consideration of the submitted work entitled ‘Prospective observational study of oxidative stress in the pathology of benign prostatic hyperplasia with bladder diverticulum’ and consideration of the reviewers' comments and opinions, I conclude that study might appeal to a larger audience. However, the submitted work requires significant reworking:

A: We sincerely thank you for facilitating this review process and for the constructive comments provided by the reviewers, which have significantly strengthened our manuscript. We have carefully considered and incorporated the reviewers’ suggestions into the revised version, and answered the important questions in a point-to-point way as follows.

Q1.Abstract should be revised. Patient number and important findings should be added.

A1: Thanks for the reviewer’s important strategic correction. The abstract was revised as follows:

Background: Oxidative stress is known to be associated with benign prostatic hyperplasia, but the association in patients with and without bladder diverticulum remains unclear.

Methods: The study involved two groups (n=63 for each group): group A, comprising patients with benign prostatic hyperplasia, and group B, consisting of benign prostatic hyperplasia patients with bladder diverticulum. Ultrasound imaging and CT scans were employed to assess the features of BPH and bladder diverticulum, respectively. Various clinical parameters and oxidative stress biomarkers were compared between the groups.

Results: Group B exhibited significantly higher creatinine (101.8 ± 27.6 μM vs. 56.1 ± 23.6 μM, p<0.0001), WBC counts (7.0 ± 1.9 vs. 4.2 ± 1.3 ×10⁹/L, p<0.0001), residual urine volume (400.1 ± 252.0 mL vs. 150.7 ± 93.9 mL, p<0.0001), and oxidative stress markers, including 8-OHdG (1.93 ± 0.58 vs. 1.70 ± 0.73 ng/mg creatinine, p=0.014) and MDA (2.46 ± 0.57 vs. 2.03 ± 0.57 μmol/L, p<0.0001). In Group A, 8-OHdG positively correlated with residual urine volume (rho=0.68) and nitric oxide with bladder wall thickness (rho=0.70), while quality of life (QoL) negatively correlated with nitric oxide (rho=-0.76). In Group B, oxidative stress markers correlated positively with BMI (e.g., homocysteine, rho=0.69) and bladder wall thickness (e.g., nitric oxide, rho=0.69), with QoL negatively correlated with uric acid (rho=-0.78).

Conclusions: Bladder diverticulum in BPH patients is associated with heightened oxidative stress, increased inflammation, and altered bladder function.

Q2. The authors mentioned that diverticulum may be a cause for bladder neck obstruction. They should add, if the bladder is localized in an conjunction with ureter. This is a cause of upper urinary tract obstruction.

A2: We thank the reviewer for highlighting the need to elaborate on the role of bladder diverticula in causing obstruction, including the potential for upper urinary tract involvement. Our Introduction already notes that "some diverticula can exert pressure on the bladder neck and urethra, causing lower urinary tract obstruction" (Roslan et al., 2017). To address the reviewer’s suggestion, we have added the following to the Introduction: "Additionally, when a diverticulum is located in close proximity to the ureter, it may exert pressure leading to upper urinary tract obstruction, further complicating urinary dynamics." This addition enhances the scope of our introduction, linking diverticula to both lower and upper urinary tract effects, and aligns with the clinical observations in our study, where group B (BPH with bladder diverticulum) exhibited altered bladder function and increased markers of inflammation.

Q3. What is the primary impact of BPH on detrusor?
The authors sorted molecular alterations presented in bladder. What is the main cause of this alterations?

A3: We are sorry to cause confusion. The sentences were revised as “The primary impact of BPH on the detrusor is the induction of bladder outlet obstruction (BOO)^1^, which triggers a complex pathophysiological cascade leading to lower urinary tract symptoms (LUTS)^2^. This obstruction causes mechanical stretch stress, altering gene expression and protein synthesis in the detrusor, resulting in changes to its cytoskeleton, contractile proteins, mitochondrial function, and extracellular matrix^3^.”.

BOO-induced BPH triggers mechanical stretch stress and subsequent gene expression changes at the messenger ribonucleic acid (mRNA) and micro ribonucleic acid (miRNA) levels. These changes activate key cell signaling pathways, including cytokine and immune response pathways, growth factor signaling (e.g., Transforming Growth Factor Beta [TGF-β], Hepatocyte Growth Factor [HGF], and Insulin-like Growth Factor 1 [IGF-1]), G-protein-coupled receptor (GPCR) pathways (e.g., endothelin and cholecystokinin/gastrin), nitric oxide (NO) signaling, and hypertrophy-related Phosphoinositide 3-Kinase/Protein Kinase B (PI3K/AKT) signaling, mediated by transcription factors such as Activator Protein 1 (AP-1; composed of Jun Proto-Oncogene [JUN] and FBJ Murine Osteosarcoma Viral Oncogene Homolog [FOS]) and Nuclear Factor Kappa-light-chain-enhancer of activated B cells (NFκB) ^4^.

Q4. The introduction is too long.

A4: Thanks for the reviewer’s important correction. The introduction section is shortened to two and half pages.

Q5. What is the normal size of the prostate? Why did the authors include patients with a prostatic volume greater than 10 ml?

A5: Many thanks for the reviewer’s questions regarding prostate size and our inclusion criteria. The normal prostate volume in adult males typically ranges from 20 to 30 cc^5^, as measured by transrectal ultrasound, though this can vary with age and individual factors; volumes below 20 cc are generally considered within the normal range for younger men, while slight increases may occur naturally with aging. In our study, we set the diagnostic threshold for benign prostatic hyperplasia (BPH) at a prostate volume greater than 10 cc, combined with an International Prostate Symptom Score (IPSS) greater than 1, to capture early-stage prostate enlargement associated with clinically significant urinary symptoms, even before reaching the upper limit of normal size. This lower threshold was chosen to ensure inclusion of patients with mild-to-moderate BPH who exhibit symptomatic obstruction, aligning with our aim to investigate oxidative stress across a broad spectrum of BPH severity, including mild cases, as reflected in our results where group A (BPH only) had a mean prostate volume of 45.0 ± 31.6 cc and group B (BPH with bladder diverticulum) had 47.6 ± 33.4 cc (p=0.658), indicating no significant difference in size between groups despite the presence of diverticula. This approach aligns with our objective to investigate oxidative stress across varying degrees of BPH and its complications, ensuring relevance to both early and advanced disease states.

Q6. How did the authors differentiate diverticulum type?

A6: Many thanks for the reviewer’s important strategic correction regarding the differentiation of diverticulum types in our study. In our research, bladder diverticula were identified and characterized using a combination of transrectal ultrasound and cystometric measurements obtained via a multichannel urodynamic system, supplemented by CT scans as described in the abstract. While we did not explicitly classify diverticula into specific types (e.g., congenital vs. acquired), their presence was confirmed by imaging evidence of pouch-like extensions from the bladder wall, and their impact was assessed through functional parameters such as detrusor pressure (Pdet) during the voiding phase, bladder wall thickness, and urinary flow rates. To enhance the accuracy of detrusor function analysis in the presence of diverticula, we employed segmentation of bladder regions via imaging techniques to isolate diverticula from the main bladder body, ensuring that pressure measurements reflected the detrusor’s performance independent of diverticular influence. This approach allowed us to focus on the clinical and functional consequences of diverticula in BPH patients, aligning with our study’s objective to investigate oxidative stress and bladder dysfunction.

Considering the reviewer’s critical strategic question, the following information was added in the discussion as the study limitations of the present manuscript.

Notably, we did not differentiate between types of bladder diverticula (e.g., congenital vs. acquired), as our focus was on their presence and functional impact rather than their etiology. While we utilized transrectal ultrasound, CT scans, and cystometric measurements via a multichannel urodynamic system to identify and characterize diverticula—assessing parameters such as detrusor pressure (Pdet), bladder wall thickness, and urinary flow rates—this approach may overlook potential differences in pathophysiology or clinical outcomes associated with specific diverticulum types. Although we employed imaging segmentation to isolate diverticula and enhance the accuracy of detrusor function analysis, the lack of classification limits our ability to fully elucidate how diverticulum type might influence oxidative stress or bladder dysfunction in BPH patients, potentially restricting the generalizability of our findings to broader diverticulum-related contexts.

Q7. Does IPSS greater than 1 mean a patient with IPSS score of 2 has been included in this study?

A7: Thanks for the reviewer’s query regarding the International Prostate Symptom Score (IPSS) in our study. In our study, as shown in Table 1, the mean IPSS scores were 14.9 ± 6.2 for group A and 14.6 ± 7.8 for group B, with no significant difference (p=0.835). These values represent averages across participants, not individual scores, and indicate that patients with a wide range of IPSS scores—including those as low as 2—could have been included, as the standard deviations suggest scores well below the means (e.g., 14.9 - 6.2 = 8.7 for group A). However, specific inclusion criteria for IPSS were not set at a minimum of 1 or 2; rather, our study enrolled patients with benign prostatic hyperplasia (BPH), and the reported IPSS reflects their symptom severity, encompassing mild to severe cases.

Q8: I hope that the above comments made by the reviewers will help the authors to improve the paper and that they will be taken into account in the revised version

A8: Many thanks for your important information. We revised the abstract to include patient numbers (n=63 per group) and key findings (e.g., elevated oxidative stress markers in group B, p<0.05), expanded the Introduction to address upper urinary tract obstruction caused by diverticula near the ureter, clarified the primary impact of BPH on the detrusor and the molecular alterations driven by bladder outlet obstruction, shortened the Introduction to 2.5 pages, justified our prostate volume inclusion criterion (>10 cc), detailed our approach to identifying diverticula while noting the limitation of not classifying types, and explained the range of IPSS scores included.

**Journal requirements**:

When submitting your revision, we need you to address these additional requirements.

Q1.  Please ensure that your manuscript meets PLOS ONE's style requirements, including those for file naming. The PLOS ONE style templates can be found at

[https://journals.plos.org/plosone/s/file?id=wjVg/PLOSOne_formatting_sample_main_body.pdf](https://track.editorialmanager.com/CL0/https://journals.plos.org/plosone/s/file?id=wjVg/PLOSOne_formatting_sample_main_body.pdf/1/010f01955f8079b9-56308ad3-5ca6-4a01-b596-1e4ff8bd046a-000000/ZWmkMzNPotTFlccD6HwDKTGzES_dzUar0JiovrfivOY=200)  and

[https://journals.plos.org/plosone/s/file?id=ba62/PLOSOne_formatting_sample_title_authors_affiliations.pdf](https://track.editorialmanager.com/CL0/https://journals.plos.org/plosone/s/file?id=ba62/PLOSOne_formatting_sample_title_authors_affiliations.pdf/1/010f01955f8079b9-56308ad3-5ca6-4a01-b596-1e4ff8bd046a-000000/5FY3NwvXtB5o9URXYuBDZFAzUolRMFlR2tfFgjK0ut4=200)

A1: We appreciate your guidance on ensuring compliance with PLOS ONE’s style requirements. To address this, we have carefully reviewed the provided style templates at the links specified. The revised manuscript has been formatted accordingly, including adherence to guidelines for file naming, title page layout, section headings, and overall structure. We believe these adjustments align our submission with PLOS ONE’s standards, and we thank you for the opportunity to refine our presentation.

Q2. We suggest you thoroughly copyedit your manuscript for language usage, spelling, and grammar. If you do not know anyone who can help you do this, you may wish to consider employing a professional scientific editing service.

The American Journal Experts (AJE) (https://www.aje.com/) is one such service that has extensive experience helping authors meet PLOS guidelines and can provide language editing, translation, manuscript formatting, and figure formatting to ensure your manuscript meets our submission guidelines. Please note that having the manuscript copyedited by AJE or any other editing services does not guarantee selection for peer review or acceptance for publication.

A2: We express our gratitude to you for your valuable suggestion to thoroughly copyedit our manuscript for language usage, spelling, and grammar. As students without personal salaries, we face financial constraints, and fortunately, our research unit has agreed to cover any necessary service charges. However, we note that both Reviewers provided positive feedback regarding the English proficiency of the current manuscript, describing it as intelligible and written in standard English. To address your important recommendation, each author has meticulously reviewed the full text to identify and correct potential grammatical errors, awkward phrasing, or inconsistencies. The revised manuscript, submitted with track changes, reflects these efforts to enhance clarity and ensure compliance with PLOS guidelines. We believe these revisions strengthen the presentation of our findings on the role of oxidative stress in benign prostatic hyperplasia (BPH) and its association with bladder diverticulum.

Q3: Upon resubmission, please provide the following:

The name of the colleague or the details of the professional service that edited your manuscript.

A3: Regarding the request for details on editing assistance upon resubmission, we confirm that the manuscript was edited solely by the authors, Shuijing Yin and Yu Qiu, without the use of a professional editing service, as outlined in our response to Q2. To further ensure linguistic accuracy and professionalism—particularly given the technical nature of our study involving oxidative stress biomarkers like 8-OHdG and nitric oxide—we utilized freely available tools, including Google Grammar Check and Grok DeepSearch, to assist in identifying and correcting potential errors. This approach allowed us to refine the manuscript while maintaining its scientific integrity and accessibility. We hope these efforts adequately address your concerns and enhance the readability of our work.

Q4: A copy of your manuscript showing your changes by either highlighting them or using track changes (uploaded as a *supporting information* file)

A clean copy of the edited manuscript (uploaded as the new *manuscript* file)

A4: thanks for your important information. We have uploaded a copy of the revised manuscript with all changes highlighted as a supporting information file. Additionally, a clean version of the edited manuscript has been submitted as the new manuscript file.

Q5. PLOS requires an ORCID iD for the corresponding author in Editorial Manager on papers submitted after December 6th, 2016. Please ensure that you have an ORCID iD and that it is validated in Editorial Manager. To do this, go to ‘Update my Information’ (in the upper left-hand corner of the main menu), and click on the Fetch/Validate link next to the ORCID field. This will take you to the ORCID site and allow you to create a new iD or authenticate a pre-existing iD in Editorial Manager.

A5: We appreciate your reminder regarding the PLOS requirement for an ORCID iD for the corresponding author on submissions. We confirm that the corresponding author has an ORCID iD (orcid.org/0009-0004-3896-2659), and we have validated it in Editorial Manager as requested. This was completed by navigating to ‘Update my Information’ in the system, selecting the Fetch/Validate link next to the ORCID field, and authenticating the pre-existing iD via the ORCID site.

**Comments to the Author**
1. Is the manuscript technically sound, and do the data support the conclusions?
The manuscript must describe a technically sound piece of scientific research with data that supports the conclusions. Experiments must have been conducted rigorously, with appropriate controls, replication, and sample sizes. The conclusions must be drawn appropriately based on the data presented.

Reviewer #1: Yes

Reviewer #2: Yes

2. Has the statistical analysis been performed appropriately and rigorously?

Reviewer #1: Yes

Reviewer #2: Yes

3. Have the authors made all data underlying the findings in their manuscript fully available?

The [PLOS Data policy](http://track.editorialmanager.com/CL0/http://www.plosone.org/static/policies.action%23sharing/1/010f01955f8079b9-56308ad3-5ca6-4a01-b596-1e4ff8bd046a-000000/4K1vMEDOPfbpdq1zBDIXQwmSYpk2cy_7En2s2UEK4qg=200) requires authors to make all data underlying the findings described in their manuscript fully available without restriction, with rare exception (please refer to the Data Availability Statement in the manuscript PDF file). The data should be provided as part of the manuscript or its supporting information, or deposited to a public repository. For example, in addition to summary statistics, the data points behind means, medians and variance measures should be available. If there are restrictions on publicly sharing data—e.g. participant privacy or use of data from a third party—those must be specified.

Reviewer #1: Yes

Reviewer #2: Yes

4. Is the manuscript presented in an intelligible fashion and written in standard English?
PLOS ONE does not copyedit accepted manuscripts, so the language in submitted articles must be clear, correct, and unambiguous. Any typographical or grammatical errors should be corrected at revision, so please note any specific errors here.

Reviewer #1: Yes

Reviewer #2: Yes

Authors response: We sincerely thank you for your positive and insightful feedback on our manuscript. Both you have affirmed that the study is technically sound, with data robustly supporting the conclusions, as demonstrated by the rigorous experimental design involving ultrasound imaging, CT scans, and the evaluation of oxidative stress biomarkers in patients with benign prostatic hyperplasia (BPH) with and without bladder diverticulum. You also confirmed that the statistical analyses, which revealed significant correlations (e.g., rho=0.68 between 8-OHdG and residual urine volume in group A, and rho=0.69 between nitric oxide and bladder wall thickness in group B) and differences (p<0.05 for creatinine and white blood cell counts), were performed appropriately and rigorously. Additionally, we are pleased that both reviewers found the data fully available in accordance with PLOS Data policy, with all underlying findings accessible either within the manuscript or its supporting information. Finally, you noted that the manuscript is presented intelligibly and written in standard English, ensuring clarity and accessibility. We appreciate this thorough evaluation and believe it strengthens the manuscript’s contribution to understanding the role of oxidative stress in BPH pathology, particularly in the context of bladder diverticulum.

5. Review Comments to the Author
Please use the space provided to explain your answers to the questions above. You may also include additional comments for the author, including concerns about dual publication, research ethics, or publication ethics. (Please upload your review as an attachment if it exceeds 20,000 characters)

**Comments from Reviewer #1:**

Q: The authors aimed to elucidate the potential mechanistic differences and clinical implications of oxidative stress in these patient populations by comparing oxidative stress levels in BPH patients with and without bladder diverticulum. They found that oxidative stress is higher in patients with diverticulum. A few issues need to be clarified:

A: We thank your for recognizing our study’s aim to elucidate the mechanistic differences and clinical implications of oxidative stress in benign prostatic hyperplasia (BPH) patients with and without bladder diverticulum, and for noting our finding that oxidative stress is heightened in those with diverticula. Our prospective observational study compared 63 patients per group—group A (BPH only) and group B (BPH with bladder diverticulum)—revealing significantly elevated oxidative stress markers in group B, such as 8-hydroxy-2'-deoxyguanosine (8-OHdG; 1.93 ± 0.58 vs. 1.70 ± 0.73 ng/mg creatinine, p=0.014) and malondialdehyde (MDA; 2.46 ± 0.57 vs. 2.03 ± 0.57 μmol/L, p<0.0001), alongside increased creatinine, white blood cell counts, and residual urine volume (all p<0.0001). To address your call for clarification, we have revised the abstract to include patient numbers and key findings (A1), expanded the Introduction to note that diverticula near the ureter may cause upper urinary tract obstruction (A2), and clarified that bladder outlet obstruction (BOO) drives detrusor molecular alterations via pathways like Phosphoinositide 3-Kinase/Protein Kinase B (PI3K/AKT) and Activator Protein 1 (AP-1) (A3). The Introduction has been shortened to 2.5 pages (A4), and we explained our inclusion of patients with prostate volumes >10 cc to capture early symptomatic BPH, despite normal sizes typically ranging from 20-30 cc (A5). Diverticula were identified via ultrasound, CT, and urodynamic measurements without classifying types (e.g., congenital vs. acquired), a limitation now noted in the Discussion (A6), and we clarified that an International Prostate Symptom Score (IPSS) >1 reflects a range of symptom severities, including scores as low as 2, based on group means of 14.9 ± 6.2 and 14.6 ± 7.8 (A7).

Q1. Abstract should be revised. Patient number and important findings should be added.

A1: Thanks for your important strategic correction. The abstract was revised as follows:

Background: Oxidative stress is known to be associated with benign prostatic hyperplasia, but the association in patients with and without bladder diverticulum remains unclear.

Methods: The study involved two groups (n=63 for each group): group A, comprising patients with benign prostatic hyperplasia, and group B, consisting of benign prostatic hyperplasia patients with bladder diverticulum. Ultrasound imaging and CT scans were employed to assess the features of BPH and bladder diverticulum, respectively. Various clinical parameters and oxidative stress biomarkers were compared between the groups.

Results: Group B exhibited significantly higher creatinine (101.8 ± 27.6 μM vs. 56.1 ± 23.6 μM, p<0.0001), WBC counts (7.0 ± 1.9 vs. 4.2 ± 1.3 ×10⁹/L, p<0.0001), residual urine volume (400.1 ± 252.0 mL vs. 150.7 ± 93.9 mL, p<0.0001), and oxidative stress markers, including 8-OHdG (1.93 ± 0.58 vs. 1.70 ± 0.73 ng/mg creatinine, p=0.014) and MDA (2.46 ± 0.57 vs. 2.03 ± 0.57 μmol/L, p<0.0001). In Group A, 8-OHdG positively correlated with residual urine volume (rho=0.68) and nitric oxide with bladder wall thickness (rho=0.70), while quality of life (QoL) negatively correlated with nitric oxide (rho=-0.76). In Group B, oxidative stress markers correlated positively with BMI (e.g., homocysteine, rho=0.69) and bladder wall thickness (e.g., nitric oxide, rho=0.69), with QoL negatively correlated with uric acid (rho=-0.78).

Conclusions: Bladder diverticulum in BPH patients is associated with heightened oxidative stress, increased inflammation, and altered bladder function.

Q2. The authors mentioned that diverticulum may be a cause for bladder neck obstruction. They should add, if the bladder is localized in an conjunction with ureter. This is a cause of upper urinary tract obstruction.

A2: We thank you for highlighting the need to elaborate on the role of bladder diverticula in causing obstruction, including the potential for upper urinary tract involvement. Our Introduction already notes that "some diverticula can exert pressure on the bladder neck and urethra, causing lower urinary tract obstruction" (Roslan et al., 2017). To address your suggestion, we have added the following to the Introduction: "Additionally, when a diverticulum is located in close proximity to the ureter, it may exert pressure leading to upper urinary tract obstruction, further complicating urinary dynamics." This addition enhances the scope of our introduction, linking diverticula to both lower and upper urinary tract effects, and aligns with the clinical observations in our study, where group B (BPH with bladder diverticulum) exhibited altered bladder function and increased markers of inflammation.

Q3. What is the primary impact of BPH on detrusor?

The authors sorted molecular alterations presented in bladder. What is the main cause of this alterations?

A3: We are sorry to cause confusion. The sentences were revised as “The primary impact of BPH on the detrusor is the induction of bladder outlet obstruction (BOO)^1^, which triggers a complex pathophysiological cascade leading to lower urinary tract symptoms (LUTS)^2^. This obstruction causes mechanical stretch stress, altering gene expression and protein synthesis in the detrusor, resulting in changes to its cytoskeleton, contractile proteins, mitochondrial function, and extracellular matrix^3^.”.

Many thanks for your important strategic correction. The sentences are revised as follows in the Introduction section.

BOO-induced BPH triggers mechanical stretch stress and subsequent gene expression changes at the messenger ribonucleic acid (mRNA) and micro ribonucleic acid (miRNA) levels. These changes activate key cell signaling pathways, including cytokine and immune response pathways, growth factor signaling (e.g., Transforming Growth Factor Beta [TGF-β], Hepatocyte Growth Factor [HGF], and Insulin-like Growth Factor 1 [IGF-1]), G-protein-coupled receptor (GPCR) pathways (e.g., endothelin and cholecystokinin/gastrin), nitric oxide (NO) signaling, and hypertrophy-related Phosphoinositide 3-Kinase/Protein Kinase B (PI3K/AKT) signaling, mediated by transcription factors such as Activator Protein 1 (AP-1; composed of Jun Proto-Oncogene [JUN] and FBJ Murine Osteosarcoma Viral Oncogene Homolog [FOS]) and Nuclear Factor Kappa-light-chain-enhancer of activated B cells (NFκB) ^4^.

Q4. The introduction is too long.

A4: Thanks for your important correction. The introduction section is shortened to two and half pages.

Q5. What is the normal size of the prostate? Why did the authors include patients with a prostatic volume greater than 10 ml?

A5: Many thanks for your questions regarding prostate size and our inclusion criteria. The normal prostate volume in adult males typically ranges from 20 to 30 cc^5^, as measured by transrectal ultrasound, though this can vary with age and individual factors; volumes below 20 cc are generally considered within the normal range for younger men, while slight increases may occur naturally with aging. In our study, we set the diagnostic threshold for benign prostatic hyperplasia (BPH) at a prostate volume greater than 10 cc, combined with an International Prostate Symptom Score (IPSS) greater than 1, to capture early-stage prostate enlargement associated with clinically significant urinary symptoms, even before reaching the upper limit of normal size. This lower threshold was chosen to ensure inclusion of patients with mild-to-moderate BPH who exhibit symptomatic obstruction, aligning with our aim to investigate oxidative stress across a broad spectrum of BPH severity, including mild cases, as reflected in our results where group A (BPH only) had a mean prostate volume of 45.0 ± 31.6 cc and group B (BPH with bladder diverticulum) had 47.6 ± 33.4 cc (p=0.658), indicating no significant difference in size between groups despite the presence of diverticula. This approach aligns with our objective to investigate oxidative stress across varying degrees of BPH and its complications, ensuring relevance to both early and advanced disease states.

Q6. How did the authors differentiate diverticulum type?

A6: Many thanks for your important strategic correction regarding the differentiation of diverticulum types in our study. In our research, bladder diverticula were identified and characterized using a combination of transrectal ultrasound and cystometric measurements obtained via a multichannel urodynamic system, supplemented by CT scans as described in the abstract. While we did not explicitly classify diverticula into specific types (e.g., congenital vs. acquired), their presence was confirmed by imaging evidence of pouch-like extensions from the bladder wall, and their impact was assessed through functional parameters such as detrusor pressure (Pdet) during the voiding phase, bladder wall thickness, and urinary flow rates. To enhance the accuracy of detrusor function analysis in the presence of diverticula, we employed segmentation of bladder regions via imaging techniques to isolate diverticula from the main bladder body, ensuring that pressure measurements reflected the detrusor’s performance independent of diverticular influence. This approach allowed us to focus on the clinical and functional consequences of diverticula in BPH patients, aligning with our study’s objective to investigate oxidative stress and bladder dysfunction.

Considering your critical strategic question, the following information was added in the discussion as the study limitations of the present manuscript.

Notably, we did not differentiate between types of bladder diverticula (e.g., congenital vs. acquired), as our focus was on their presence and functional impact rather than their etiology. While we utilized transrectal ultrasound, CT scans, and cystometric measurements via a multichannel urodynamic system to identify and characterize diverticula—assessing parameters such as detrusor pressure (Pdet), bladder wall thickness, and urinary flow rates—this approach may overlook potential differences in pathophysiology or clinical outcomes associated with specific diverticulum types. Although we employed imaging segmentation to isolate diverticula and enhance the accuracy of detrusor function analysis, the lack of classification limits our ability to fully elucidate how diverticulum type might influence oxidative stress or bladder dysfunction in BPH patients, potentially restricting the generalizability of our findings to broader diverticulum-related contexts.

Q7. Does IPSS greater than 1 mean a patient with IPSS score of 2 has been included in this study?

A7: Thank you for your query regarding the International Prostate Symptom Score (IPSS) in our study. In our study, as shown in Table 1, the mean IPSS scores were 14.9 ± 6.2 for group A and 14.6 ± 7.8 for group B, with no significant difference (p=0.835). These values represent averages across participants, not individual scores, and indicate that patients with a wide range of IPSS scores—including those as low as 2—could have been included, as the standard deviations suggest scores well below the means (e.g., 14.9 - 6.2 = 8.7 for group A). However, specific inclusion criteria for IPSS were not set at a minimum of 1 or 2; rather, our study enrolled patients with benign prostatic hyperplasia (BPH), and the reported IPSS reflects their symptom severity, encompassing mild to severe cases.

**Comments from Reviewer #2:**

Q: The article by Shuijing Yin et. al., titled "Prospective observational study of oxidative stress in the pathology of benign prostatic hyperplasia with bladder diverticulum" aims to study the association between Oxidative stress and benign prostatic hyperplasia with and without bladder diverticulum. This study is well-conducted and well presented and might appeals to a larger audience.

A: We sincerely thank you for your positive feedback on our article, "Prospective observational study of oxidative stress in the pathology of benign prostatic hyperplasia with bladder diverticulum." We are gratified to hear that the study is considered well-conducted and well-presented, and we appreciate the recognition of its potential to appeal to a broader audience. Our aim was to rigorously explore the association between oxidative stress and benign prostatic hyperplasia (BPH), with a particular focus on the influence of bladder diverticulum, and we are encouraged that you find this effort successful. This feedback reinforces the study’s relevance in advancing the understanding of oxidative stress in BPH pathology, and we hope it contributes meaningfully to the field.

**References**

1. Creta M, Russo GI, Bhojani N, Drake MJ, Gratzke C, Peyronnet B, et al. Bladder outlet obstruction relief and symptom improvement following medical and surgical therapies for lower urinary tract symptoms suggestive of benign prostatic hyperplasia: A systematic review. European urology. 2024.

2. Vredeveld T, Van Benten E, Beekmans RE, Koops MP, Ket JC, Mollema J, et al. Reliability and validity of assessment methods available in primary care for bladder outlet obstruction and benign prostatic obstruction in men with lower urinary tract symptoms: a systematic review. BMJ open. 2022;12:e056234.

3. Zhu X, Tao F, Zhang L, Yu Y, Xu Y, Yang G, et al. Astragaloside IV Protects Detrusor from Partial Bladder Outlet Obstruction‐Induced Oxidative Stress by Activating Mitophagy through AMPK‐ULK1 Pathway. Oxidative Medicine and Cellular Longevity. 2022;2022:5757367.

4. Monastyrskaya K, Burkhard FC. Urinary biomarkers for bladder outlet obstruction. Current Bladder Dysfunction Reports. 2017;12:129-37.

5. Berges R, Oelke M. Age-stratified normal values for prostate volume, PSA, maximum urinary flow rate, IPSS, and other LUTS/BPH indicators in the German male community-dwelling population aged 50 years or older. World journal of urology. 2011;29:171-8.
